# A mixture of attention experts-embedded flow-based generative model to create synthetic cells in single-cell RNA-Seq datasets

**DOI:** 10.1371/journal.pcbi.1013525

**Published:** 2025-10-06

**Authors:** Sultan Sevgi Turgut Ögme, Nizamettin Aydin, Zeyneb Kurt

**Affiliations:** 1 Department of Computer Engineering, Yildiz Technical University, Istanbul, Türkiye; 2 Department of Computer Engineering, Istanbul Technical University, Istanbul, Türkiye; 3 Information School, The University of Sheffield, The Wave, Sheffield, United Kingdom; University of Pittsburgh, UNITED STATES OF AMERICA

## Abstract

Single-cell RNA-seq (scRNAseq) analyses performed at the cellular level aim to understand the cellular landscape of tissue sections, offer insights into rare cell-types, and identify marker genes for annotating distinct cell types. ScRNAseq analyses are widely applied to cancer research to understand tumor heterogeneity, disease progression, and resistance to therapy. Single-cell data processing is a challenging task due to its high-dimensionality, sparsity, and having imbalanced class(cell-type) distributions. An accurate cell-type identification is highly dependent on preprocessing and quality control steps. To address these issues, generative models have been widely used in recent years. Techniques frequently used include Variational Autoencoders (VAE), Generative Adversarial Networks (GANs), Gaussian-based methods, and, more recently, Flow-based (FB) generative models. We developed a Masked Affine Autoregressive transform-embedded FB (MAF-FB) model. Then, to improve MAF-FB further, we incorporated a mixture of experts (MOE) of attention mechanisms on top of it, resulting in our proposed MOE-FB model. We conducted a comparative analysis of fundamental generative models, aiming to serve as a preliminary guidance for developing novel automated scRNAseq data analysis systems. We performed a large-scale analysis by combiningfour datasets derived from pancreatic tissue sections and for further generalizability assessments, we employed Peripheral Blood Mononuclear Cells (PBMC68K and PBMC3K) and Human Cell Atlas Bone Marrow (HCA-BM10K) datasets. We utilized VAE, GAN, Gaussian Copula, and Automated Cell-Type-informed Introspective Variational Autoencoder (ACTIVA), and compared them against our two novel FB models, MAF-FB and MOE-FB for ScRnaseq synthesis. To evaluate the performances of generative models, we used various discrepancy metrics and performed automated cell-type classification tasks. We also identified differentially expressed genes for each cell type, and inferred cell-cell interactions based on ligand-receptor bindings across distinct cell-type pairs. Among the generative models, FB models, especially MOE-FB, consistently outperformed others across all experimental setups in both discrepancy metrics with comparison to the baseline test set and cell-type classification tasks (with an F1-score of 0.90 precision of 0.89 and recall of 0.92 for the integrated pancreatic datasets). MOE-FB produced biologically more relevant synthetic data, and ligand–receptor–based cell–cell interactions inferred from the synthetic cells closely resemble the original data, achieving an RMSE of 0.65 against the corresponding pancreatic test set. These findings highlight the potential and promising use of FB models, especially MOE-FB, in scRNAseq analyses.

## Author summary

Single-cell RNA sequencing (scRNA-seq) analyses focus on identifying distinct cell types and marker genes. Traditional methods face challenges with high dimensionality, sparsity, and sample size imbalances across cell types, limiting automated and unbiased cell-type identification. Generative AI models address these issues by generating synthetic cells for under-represented cell types, preserving biological and contextual relevance, and employing embedding mechanisms to reduce sparsity and dimensionality. We proposed a Flow Based (MAF-FB) model with Masked Affine Autoregressive transform for single-cell synthesis and a new framework that extends vanilla MAF-FB combined with a mixture of experts of attention mechanism (MOE-FB). We compared widely used generative models (Variational Autoencoders, GANs, Gaussian Copula, and ACTIVA with FB models) using integrated pancreatic and additionally external datasets. Synthetic data quality was assessed via multiple discrepancy metrics, a cell type classification task using a Random Forest model, and a ligand-receptor interaction inference task. The FB models, especially MOE-FB showed the highest potential for creating similar and biologically accurate scRNA-seq profiles to the original data. We presented a guideline for automated cell-type identification systems by addressing gaps in single-cell analysis characteristics through the integration of widely used computational biology datasets and implementation of generative models (including a vanilla and a novel, FB model, MAF-FB and MOE-FB frameworks, respectively).

## 1. Introduction

Single-cell RNA-sequencing (scRNAseq) analysis aims to identify different cell types that make up tissues or tumour microenvironments [1] as well as the marker genes that can distinguish particular cell types from others. Various software platforms and tools have been presented for the implementation and evaluation of these analyses [2,3]. Cell type and marker gene determination usually needs manual operations and is quite time-consuming. Therefore, in recent years, emphasis has been placed on automating these steps.

Among the analysis tools, Seurat [2] and Single-Cell-Experiment (SCE) [3] R packages stand out and their workflows are similar. Both workflows include; quality control, normalization, feature selection, cell integration, dimension reduction, and visualization steps. After the pre-processing steps, a clustering method (e.g., Kmeans) groups similar cells, and Differential Gene Expression (DGE) is identified for the annotation of cell types. Especially for the annotation purposes, time-demanding work is required in addition to various analyses including manually comparing differentially expressed genes in each cell cluster. So, researchers focused on automation of cell-type identification with low error rates. Comprehensive comparative studies have been conducted for this purpose using machine learning models such as Random Forest, Support Vector Machines, Multi-Layer Perceptrons, and K-Nearest Neighborsby utilizing datasets from differing species (human, mouse) [4,5], different technologies [6], with varying sizes, and complexity.

Pre-processing steps have significant effects on the downstream processes, i.e., the identification of cell-types and marker genes. Hence in recent years, researchers have been developing frameworks to improve pre-processing steps. Single-cell data consists of large-scale and sparse matrix structures that are computationally challenging to process. Hence the pre-processing step is crucial in terms of affecting the processing time and accuracy of the downstream estimations made. Another problem is the imbalanced sample-sizedistribution across different cell clusters. Since the validation of findings needs to be carried out in high-cost laboratory environments that contributes to the class imbalance issue, the number of publicly available datasets with annotated and labeled cell types is quite limited [7]. Generative models can address the class imbalance problem by generating synthetic cells, while their embedding mechanisms effectively mitigate the sparsity and high-dimensionality issues [8].

Some studies have utilized the latent space representations of generative models to address the sparsity by reducing the dimensionality. Gronbech et al. [9] proposed a Variational Autoencoder (VAE) based single cell VAE (scVAE) method. This method skips a precise data preprocessing step by using the original count matrix as its input and offers a model that can reliably estimate the representations of cells in the latent space. The Gaussian Mixture method, proposed by Choi et al. [10] obtained embedding representations by using the VAE method on both cell and gene basis. Gene-gene relationships and hub genes were identified by creating gene-based embedding representations. On the other hand, generative models have been investigated to address the issues around imbalanced class distributions by generating synthetic cells. Marouf et al. [11] proposed the scGAN model using Generative Adversarial Networks (GANs) for a realistic generation of scRNAseq data. Their model learns non-linear gene-gene dependencies from complex, multi-cell type samples and uses this information to generate synthetic cells. Yu et al. [12] introduced a novel method called MichiGAN, which combines the strengths of VAE and GAN models. This deep generative model performs sampling using representations that semantically manipulate cells without compromising data quality. Heydari et al. [13] presented the Automated Cell-Type-informed Introspective Variational Autoencoder (ACTIVA) model, which utilizes conditional VAE conditioning on cell-type information during the cell generation process. It consists of three networks; an encoder which is employed as a discriminator distinguishing synthetic cells from real ones, a decoder that works as a generative network creating synthetic data, and a cell-type classification network. ACTIVA demonstrates superior performance compared to solely GAN-based models by generating more realistic synthetic cells. Palma et al. proposed a flow-based generative model named cellFlow [14] utilizing Conditional Flow Matching [15]. This model is based on a likelihood model and negative binomial distribution. The authors highlighted the importance of using raw counts for synthetic cell generation and downstream analysis. They compared the performance of several generative models to cellFlow, finding that cellFlow achieves results comparable to existing methods.

In summary, the structure of single-cell data has urged researchers to adopt generative models into their work. In the field of single-cell RNA-seq, various versions and workflows of GAN and VAE models have been extensively investigated. In contrast, flow-based models are relatively recent and have not been sufficiently developed for single-cell applications, offering significant potential for further research. Therefore, in this study, we use a Masked Autoregressive Flow-based model (MAF-FB) as a baseline, to the best of our knowledge, this transformation has not previously been applied in single-cell RNA-seq analysis. We further extend this architecture by incorporating a Mixture-of-Experts (MoE) attention mechanism and learnable feature masking, resulting in a novel model (MOE-FB) specifically designed for single-cell data generation. Furthermore, generative models are predominantly applied to image and text analysis, so we present a comparative evaluation using GAN, VAE, and Gaussian Copula models from the Synthetic Data Vault (SDV) library, originally designed for tabular data, along with our flow-based approaches.

## 2. Results

**Fig 1:**
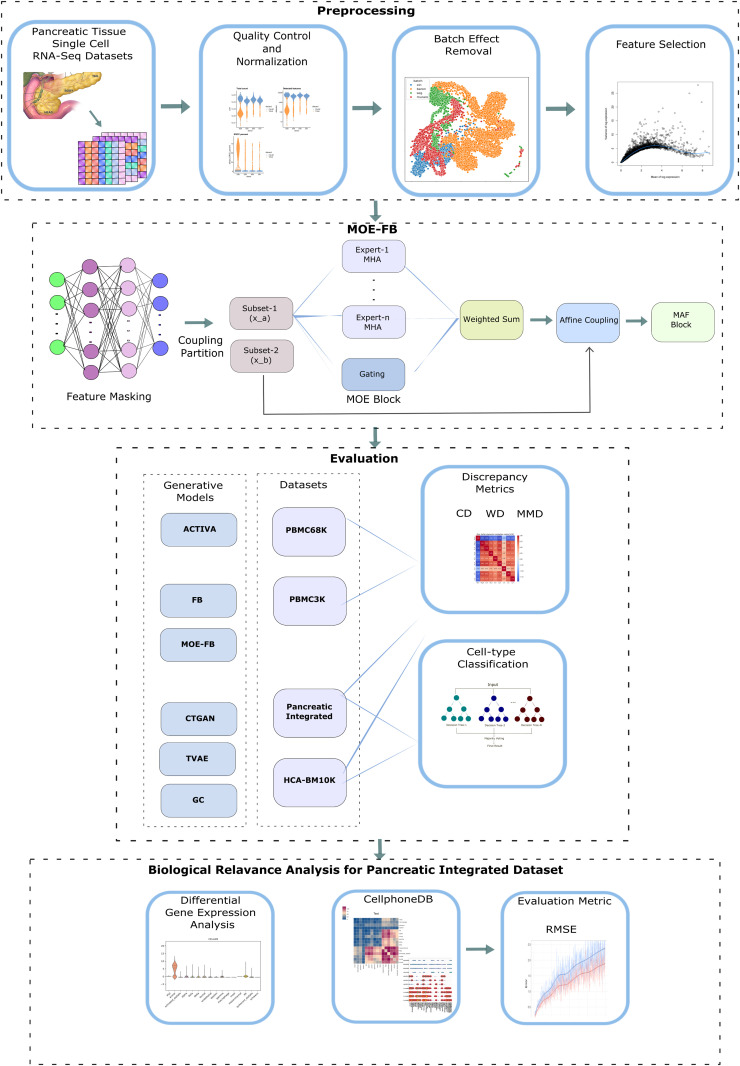
Overall workflow diagram.

Fig 1 presents our study’s overall framework. Our workflow consists of pancreatic tissue dataset curation, data preprocessing, proposed model framework, synthetic cell generation, benchmarking with different generative models and external datasets, evaluation with discrepancy metrics and cell-type classification. It also incorporates marker gene identification and cell-cell interaction analysis as key components in downstream analyses.

### 2.1. Datasets

To comprehensively assess model performances across diverse biological contexts, we selected four distinct single-cell RNA-seq datasets representing different tissue types and experimental conditions.

#### 2.1.1. Integrated pancreatic tissue dataset.

**Fig 2:**
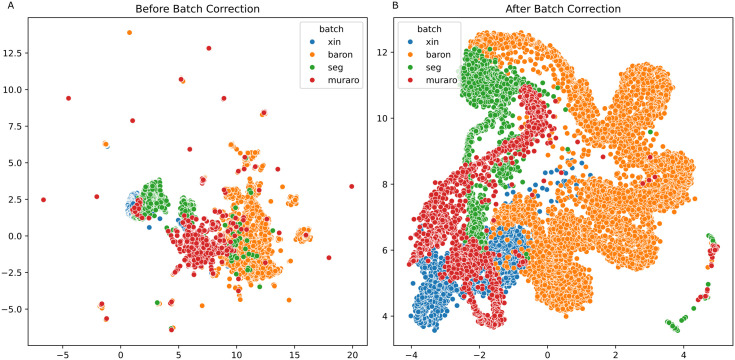
Batch Effect Correction. (A) Before (B) After correction.

We first created an integrated pancreatic single-cell RNA-seq dataset comprising a total of 14,333 cells by combining four publicly available pancreatic tissue datasets (see Materials and Methods). Since datasets are curated from different resources, their integration may cause an unintentional bias introduced by the batch effect. Hence, they need to undergo a batch effect correction process. Fig 2 demonstrates the spread of the integrated pancreatic data before and after the batch correction was performed. Before the batch effect removal, cells obtained from different studies appear separately from one another. But after the batch effect correction procedure, they are more homogeneously distributed on the 2D UMAP plane, as represented by the first two principal components of the cells. To assess the effectiveness of batch effect correction, we computed the scaled average silhouette batch score from [16], which subtracts the absolute silhouette score from 1 before averaging over features. The ultimate score ranges from 0 to 1, with values closer to 1 indicating reduced batch effects. The silhouette value before correction is 0.8876, while after the correction it becomes 0.9437, indicating improved and sensible dataset integration. To enhance robustness and generalizability, we employed a 5-fold cross-validation strategy, splitting the data into 80% training and 20% testing in each fold. To ensure consistency across folds, we selected a set of 3,000 highly variable genes (HVGs) and used the same number of genes for all folds. As a result, 3000 genes out of 15839 were selected and the final large-scale analysis data has a size of 11338 (cells) x 3000(genes) for training, 2834 (cells) x 3000 (genes) for testing in each fold.

#### 2.1.2. Peripheral blood mononuclear cells (PBMC).

To enable a direct comparison with the state-of-the-art model ACTIVA, we used a well-known scRNAseq dataset, known as peripheral blood mononuclear cells (PBMCs). This dataset contains 68,579 PBMC cells collected from a healthy donor [17] and, after preprocessing, includes 17,789 genes, 61,588 cells for training and 6,991 cells for testing. Owing to its diverse cell types, substantial complexity, and large sample size, the PBMC68K dataset serves as a robust benchmark for evaluating generative modeling approaches.

In addition, for running all our models, we used a subset of the PBMC (PBMC3K) dataset containing 2700 cells and 2,000 highly variable genes (HVGs). SDV library models could not handle the high-dimensional data and didn’t support data loaders for dividing the data into chunks, so we executed and tested this subset data to compare all models.

#### 2.1.3. Human cell atlas human bone marrow (HCA-BM).

We collected immune cells from the bone marrow scRNA-seq dataset on the Human Cell Atlas (HCA-BM), which consisted of approximately 380,000 cells. SDV library models’ restricts the use of the entire dataset. To enable execution and comparison of all generative models, we created a subset containing 10,000 cells and 3,000 highly variable genes (HVGs), and referred to it as HCA-BM10K (see the subsampling procedure in Materials and Methods). We applied a 5-fold cross-validation strategy, splitting the data into 80% training and 20% testing in each fold.

### 2.2. Generative models

In this study, we developed flow-based generative models, which have recently gained attention in the field. We present two main variants of FB models: (1) a baseline flow-based model incorporating the Masked Autoregressive Flow (MAF) transformation (vanilla MAF-FB), and (2) an enhanced version integrating a Mixture-of-Experts attention mechanism with baseline MAF-FB (MOE-FB). To provide a comprehensive comparative analysis, we additionally employed three tabular generative models from the SDV library, CTGAN, TVAE, and Gaussian Copula (GC), which are widely used for structured, tabular data generation. Furthermore, we included the state-of-the-art ACTIVA model [13] in our experiments to benchmark our approaches against a leading method in single-cell RNA-seq data generation. We generated the same number of samples as in the test set for PBMC3K and PBMC68K without considering cell-type labels to compare with ACTIVA model.

**Fig 3:**
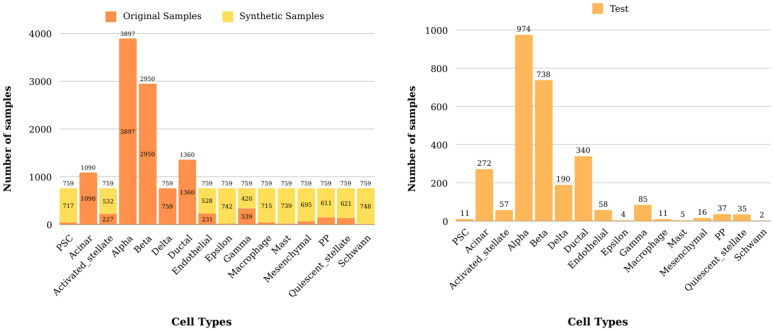
Distribution of cell counts across distinct cell types for the first fold of integrated pancreatic dataset.

For pancreatic and HCA-BM10K datasets, the third quartile (Q3) value of cell numbers across all different cell types in a given training dataset was used as the optimal number of synthetic samples to be generated (see Materials and Methods). As an example, Fig 3 shows the number of original train samples, synthetic samples and test sets across distinct cell types for the first fold of integrated pancreatic dataset.

### 2.3. Evaluation of generative models and cell type classification

In line with previous studies [11,13,14], which commonly assess the quality of synthetic data generated by generative models using the first 50 principal components (PC-50), we also employed PCA and used the PC-50 representation in our evaluation. Generative models should preserve both biological and contextual patterns present in original data points and retain the relationship motives that exist between original features. So, we evaluated generative models using metrics: Correlation Discrepancy (CD), Wasserstein-distance (WD), and linear Maximum Mean Discrepancy (MMD) and analyzed the differences between the original and generative sets. The metrics are described in the *Metarials and Methods* section along with their corresponding equations. For comparison, these metrics were calculated between the original training and testing set cells and these values were used as a baseline while assessing the performance of the generative models.. We used unseen test set samples and assessed their similarity to the original training samples, then we used these (dis)similarity scoresto assess which generative model demonstrates better generalizability and is able to generate more realistic synthetic data. We trained the model on the PBMC3K dataset and generated synthetic samples matching the size of the test set. The same procedure was applied to all other generative models, and the results are summarized in Table 1. For the WD and CD metrics, the MOE-FB model achieved the best performance, while flow-based models outperformed others across all three metrics. The main limitation of the SDV library’s generative models is their inability to handle high-dimensional data. Moreover, since these models do not support the data loader, the dataset cannot be split for training. Although the MOE-FB model operates efficiently, its attention mechanism leads to substantial memory usage as the number of features increases, exceeding RAM capacity when applied with 17,000 genes. Therefore, the PBMC68K dataset was evaluated only using the MAF-FB and ACTIVA methods. ACTIVA provides a pretrained PBMC68K model in its repository, which was used to generate synthetic samples matching the size of the test set. Table 2 presents the comparison results for PBMC68K, where MAF-FB outperforms across all metrics.

**Table 1:** Evaluation metrics values between the synthetic and test data for PBMC3K.

	PMC3K						
	Original Train vs Test	MOE-FB	MAF-FB	ACTIVA	CTGAN	TVAE	GC
WD↓	0.45406	**0.40528**	0.55452	0.91830	0.44015	1.24546	11.72473
CD↓	0.06149	**0.07874**	0.08153	0.32516	0.08144	0.32843	0.11055
MMD↓	0.003416	0.030967	**0.02216**	0.105935	0.060532	0.325968	0.084370

**Table 2:** Evaluation metrics values between the synthetic and test data for PBMC68K.

	PBMC68K		
	Original Train vs Test	MAF-FB	ACTIVA
WD↓	0.10384	**0.43555**	0.79137
CD↓	0.15787	**0.16031**	0.77847
MMD↓	0.00031	**0.028707**	0.055826

For the Integrated Pancreatic and HCA-BM10K datasets, synthetic samples were generated to balance the distribution of cell types. The classification performance of these balanced datasets was then evaluated. Cell type classification was performed using the Random Forest (RF) classification model. We trained the RF with solely original data (i.e., real cells) then by pooling them with synthetic cells that are generated by different generative models. Afterwards, the RF model was evaluated on a separate test set, which was not used for synthetic data generation to avoid any data leakage, for each experimental setup. Table 3 presents the classification results obtained from all models, using the selected highly variable 3000 genes. For the synthetic data generation metrics (e.g., WD), the PC-50 representation was used for comparison in line with the relevant studies. The best-performing results are highlighted in bold. In the classification results, the MOE-FB model demonstrated a clear advantage. Considering the other metrics, MAF-FB performed better for the CD metric in the HCA-BM10K dataset, although its results were very close to those of MOE-FB. In metrics where MOE-FB did not achieve the best performance, the MAF-FB model, another flow-based approach, stood out. For the integrated pancreatic dataset, while the MMD metric was highest for GC, this model performed considerably worse in the other metrics. Among the SDV models, CTGAN was the closest to the two FB models, leading in the WD metric for the HCA-BM10K dataset.

**Table 3:** Evaluation metrics values between the balanced train(original and synthetic) and test data for integrated pancreatic and HCA-BM10K datasets.

	Original Train vs Test	MOE-FB	MAF-FB	CTGAN	TVAE	GC
	Integrated Pancreatic Data (Averaged 5-Fold)					
WD↓	0.13731	**0.59546**	0.62089	0.60276	0.70711	0.77126
CD↓	0.01766	0.07132	**0.06762**	0.07760	0.09673	0.12429
MMD↓	0.000515	0.003618	0.002707	0.00431	0.005857	**0.002447**
Precision	0.8203	**0.9221**	0.8905	0.8900	0.9057	0.8469
Recall	0.7819	**0.8935**	0.8903	0.8551	0.8316	0.8068
F1	0.7984	**0.9014**	0.8837	0.8667	0.8549	0.8208
	HCA Bone Marrow (HCA-BM10K) Data (Averaged 5-Fold)					
WD↓	0.21920	0.86890	0.87103	**0.46898**	0.52454	2.73766
CD↓	0.02718	0.05946	**0.059417**	0.063497	0.07148	0.34992
MMD↓	0.001047	**0.005317**	0.005330	0.006268	0.009280	0.006166
Precision	0.9311	0.9308	0.9301	0.9267	0.9287	**0.9332**
Recall	0.8483	**0.8802**	0.8800	0.8614	0.8478	0.8463
F1	0.8765	**0.8965**	0.8964	0.8829	0.8759	0.8759

### 2.4. Visual comparison

**Fig 4:**
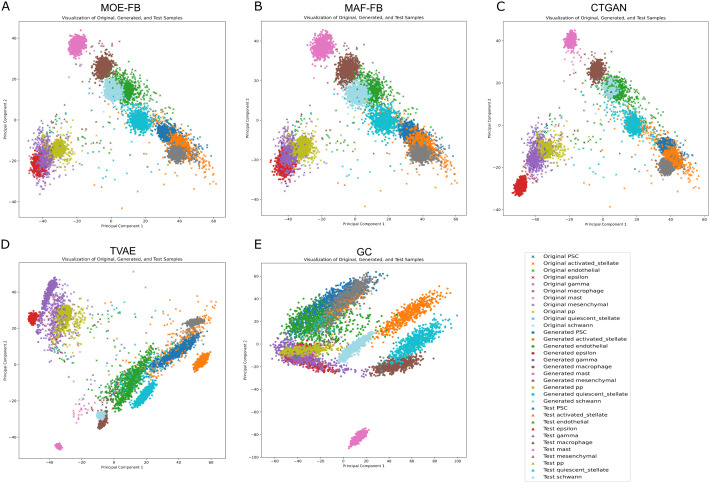
Distribution of original training, generated (synthetic) and test cells in 2-dimensional space where data generation is done by(A) MOE-FB, (B) MAF-FB, (C) CTGAN, (D) TVAE, (E) GC.

The original training samples, test samples, and generated cells (created solely from training samples) were embedded in a 2-dimensional space using PCA. Since the evaluation discrepancy metrics were computed using the PC-50 representation, we applied PCA to generate the 2D plots for consistency. The distribution of samples (original cells plus synthetic cells generated using different generative models) from the first fold of the Integrated Pancreatic dataset is shown on a 2-D scatter plot in Fig 4.

### 2.5. Differentially expressed genes (DEG) identification

**Fig 5:**
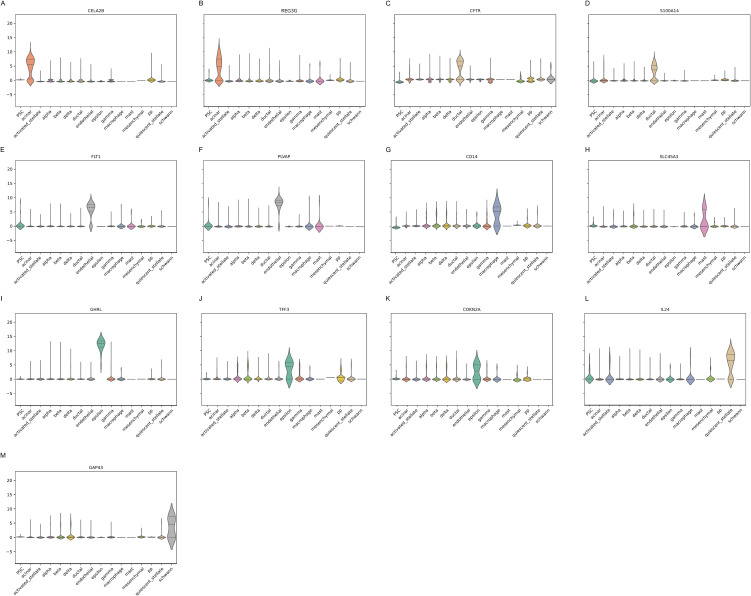
Example of mutually exclusive DEGs found in literature.

Differential Gene Expression Analysis was performed through integrating real training and test samples of integrated pancreatic dataset. DEGs that are mutually exclusive with other cell type categories were listed as candidate marker genes [18,19]. We identified the top 20 genes and retained those that were unique to each cell type, the final list of DEGs was given in S1 Table including adjusted p-value and logFC values. For each cell type, violin plots of the top significant and unique DEGs were generated. Representative examples of the generated violin plots are shown in Fig 5. We conducted a literature review to explore previous studies that validated any of the genes listed among the top 20 DEGs using resources such as DisGeNET [20,21], GeneCards [22,23], and a PubMed search. Some examples of mutually exclusive DEGs that are found in integrated pancreatic dataset, relevant cell-type, and associated with tumour include: CELA2B [24], REG3G [25,26], CFTR [27], S100A14 [28], FLT1 [29], PVALP [30,31], GHRL [32], TFF3 [33,34], CDKN2A [35], CD14 [36], SLC45A3 [37,38], IL24 [39], GAP43 [40].

### 2.6. Inferring cell-cell interactions

**Fig 6:**
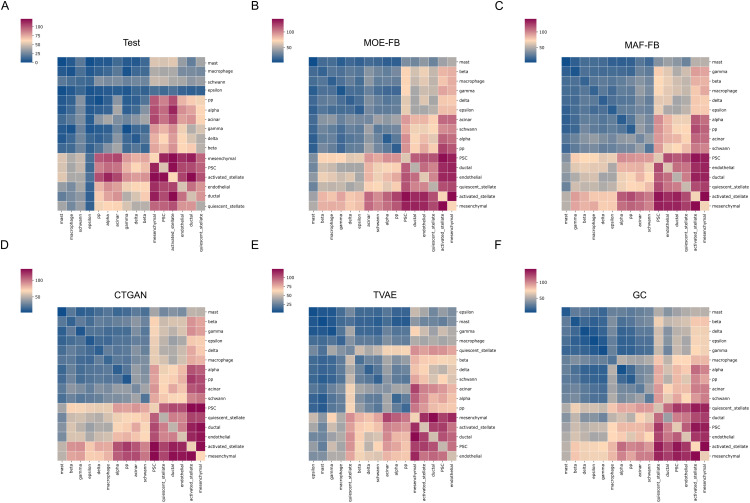
Total number of significant interactions between cell types for (A) Test set, (B) MOE-FB, (C) MAF-FB, (D) TVAE, (E) CTGAN, (F) GC.

For inferring cell-cell interactions through identifying potential ligand-receptor communication pairs, CellPhoneDB tool is frequently preferred [41,42]. We investigated the cell-cell interactions in the test, and generative models data in the first fold. We applied statistical analysis and used p-values output (S1 Appendix) generated with CellPhoneDB to construct the heatmaps. Fig 6 presents heatmaps illustrating the total number of significant cell-cell interactions that exist between different cell types. In the heatmaps, warmer colors (red) indicate a higher number of significant interactions, and cooler colors (blue) represent fewer or no significant interactions.

The heatmap of cell-cell interaction for the test data (Fig 6A), with strong interaction networks observed among stromal-associated cell types, particularly between quiescent stellate, activated stellate, ductal, endothelial, and Pluripotent stem cells (PSC) cells, suggesting active communication within the tissue microenvironment. Mesenchymal cells also exhibit moderate interactions with both stromal and certain endocrine cell types. In contrast, endocrine populations such as beta, delta, and gamma cells, and immune-associated populations such as macrophages, mast cells, and schwann cells, exhibit less significant interactions, indicating limited signaling in this context.

Heatmaps for the original cells and cells generated by the other generative models (Fig 6B-F) have higher numbers of interactions due to a higher sample size.

Table 4 presents the evaluation of generative models based on the mean expression levels (S2 Appendix) of ligand–receptor pairs for each cell–cell interaction. We compared each model with the corresponding test set values using Root Mean Squared Error (RMSE) as an evaluation metric. The MOE-FB model achieved the best performance through the lowest error values among the generative approaches, indicating the highest similarity to the test set interactions. GC showed the largest deviations, while CTGAN and TVAE yielded comparable but higher errors than the FB models.

**Table 4:** Comparison of generative models based on average expression levels of cell–cell interactions.

	Integrated Pancreatic Dataset (First Fold)					
	Original Train vs Test	MOE-FB	MAF-FB	CTGAN	TVAE	GC
RMSE↓	0.4253	**0.6522**	0.6760	0.7254	0.7251	0.8740

**Fig 7:**
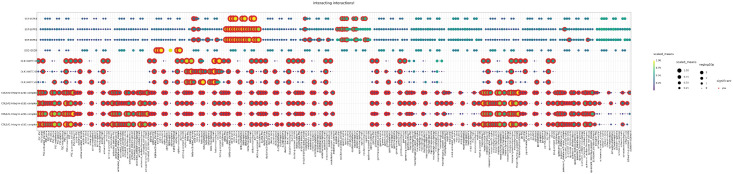
Ligand-receptor pairs representing the cell-cell interactions for the test set of integrated pancreatic dataset.

Additionally, we discovered ligand-receptor pairs, representing the cell-cell crosstalks, for the test set and generative models. Fig 7 demonstrates ligands, receptors and their interactions for the test set samples. We used the top first most significant DEGs of all cell types for plotting. Due to the large number of interactions between cells, we are unable to show all of the potential ligand-receptor pairs that are identified on a single dotplot. The X-axis refers to cell-cell interaction pairs, the y-axis refers to ligand-receptor pairs. The size of the dots reflects the percentage of the cells in interaction for the corresponding cell types, and the color of the dots, changing from blue to yellow, represents the average of the mean expression value of ligand-receptor pairs in corresponding cells (yellow represents higher expressions). The red outline indicates statistically significant (p < 0.05) ligand-receptor interaction observations. Therefore, it can be assumed that dots with a statistically significant (red outline) representation and a high-valued (yellow) expression are the strongest and most significant interactions.

Fig 7 can be summarized as follows;

Ligands correspond to cell type 1, while receptors correspond to cell type 2. First, we listed the cell types associated with the ligands and then identified significantly observed and high-valued (red outline and yellow dots) pairs. Several ligand–receptor pairs show strong and recurrent interaction patterns across multiple cell-type pairs. In particular, strong and frequent interactions between multiple stromal-associated cell type pairs are observed for the COL1A1/COL5A2-integrin α1β1/α2β1 complex, highlighting extensive extracellular matrix and adhesion signaling that supports tissue organization and intercellular communication. In the DLK1–NOTCH family interactions, beta-cells act predominantly as the ligand-producing population, while many other cell types express the corresponding NOTCH1/2/3 receptors. This pattern suggests that beta-cells are actively involved in Notch-mediated signaling with various neighboring cell populations. SST–SSTR1/2/3, generally occur in more restricted but consistent patterns in endocrine-associated(e.g., delta cell-type) interactions. Taken together, these patterns reveal both widespread and cell-type-specific communication pathways present in the dataset.

Dot plots of generative models (S3 Appendix) are similar to those of the test set. Generative models have a higher number of interactions overall. However, the significant and high-valued cell-type interaction pairs remain consistent with the test. We observed that the heatmaps shown in Fig 6 are in line with dot plots representing the strong interactions between cell types PSC, mesenchymal, and activated stellate cells, and so on.

## 3. Conclusions and discussions

Preprocessing steps in scRNAseq analysis are crucial for identifying cell types, because single cell data is sparse, has large-dimensionality and imbalanced class distributions. Although there are various publicly available scRNAseq datasets in the literature, certain cell types are under-represented and form a minority category, so especially the automated identification of these cell groups is challenging. To address these issues, generative models have been proposed and used in recent years. The VAE model has been widely employed in several studies [9,13]. Additionally, GANs and Gaussian-based models have also been explored [11]. Flow-based models have very recently been applied to single-cell analysis studies and indicated a promising potential. As a baseline, we included a flow-based model employing the Masked Autoregressive Flow transformation (MAF-FB). We also proposed a novel flow-based model (MOE-FB) that integrates a learnable masking component with a Mixture-of-Experts attention mechanism, offering a new approach for single-cell data generation. In addition, we conducted a comparative study within a well-defined workflow using the most prominent and promising generative models, including CTGAN, TVAE, and Gaussian Copula (GC) from the SDV library, as well as the state-of-the-art ACTIVA model.

Our aim was to provide a preliminary pilot study that can serve as a reference guidance for developing new, more robust, and automated cell type identification workflows that benefit from generative models to address class imbalance, sparsity, and high-dimensionality issues.

We conducted a large-scale analysis by merging commonly used four pancreatic tissue scRNAseq datasets [4–6,43] including cell type annotations, which has been rarely examined for generative models. In addition, we incorporated a 10,000-cell subset of the Human Cell Atlas Bone Marrow dataset (HCA-BM10K), generated by preserving the original cell type distributions to perform parallel analyses with the pancreatic data. We also included Peripheral Blood Mononuclear Cell datasets, namely PBMC68K (68,000 cells) and PBMC3K (~3000 cells) in our analyses to enable direct comparison with the state-of-the-art ACTIVA model. For the Pancreatic and HCA-BM10K datasets, we used generative models to balance the number of samples across different classes by increasing the sample sizes of minority classes. Synthetic cells generated by each generative model were combined with the training data. The purpose of generative models is to produce synthetic data which is similar to the original one. For the PBMC3K and PBMC68K datasets, we followed the approach used in studies such as ACTIVA,building generative models on the training data and generating synthetic samples in quantities matching those in the test set. In addition, we performed Differential Gene Expression (DEG) and cell–cell interaction analyses on our primary dataset, the integrated pancreatic dataset specifically constructed for this study. Correlation Discrepancy (CD), Wasserstein distance (WD) and Maximum Mean Discrepancy (MMD) metrics were used for measuring the discrepancy between train vs test set (as a ground-truth) samples and train set including generated (synthetic) samples vs test set samples. A smaller metric value indicates a higher similarity. The RF, which is a supervised classification model, was trained for cell type classification. Trained models were tested on a previously unseen test dataset.

For the PMC3K dataset, our proposed MOE-FB model consistently outperformed other generative models across most evaluation metrics. In terms of WD, which measures distributional similarity between real and synthetic data, MOE-FB achieved the lowest (i.e., best) score (0.40528). Correlation Discrepancy (CD)metric evaluates preservation of gene–gene correlation structures, MOE-FB again demonstrated a competitive CD performance (0.07874). The Maximum Mean Discrepancy (MMD) results further highlight MOE-FB’s advantage. With an MMD value of 0.030967, it surpassed all models except MAF-FB (0.02216). Flow-based models performed superiorly, with the proposed MOE-FB achieving the best overall results among all methods and markedly outperforming ACTIVA across all metrics. For the PBMC68K dataset, the evaluation encountered some computational constraints. Due to the high dimensionality of this dataset, the methods in the SDV library could not be executed, as they do not support data loaders, and we could not utilize data chunks. Additionally, while our proposed MOE-FB model demonstrates strong performance on other datasets, it has a known limitation: the Mixture-of-Experts module operates on a feature-wise basis, causing memory usage to increase significantly with the number of features. This leads to excessive computational demands on large datasets, making it infeasible to generate within our available resources.

Given these constraints, we compare our baseline flow-based model (MAF-FB) and the state-of-the-art ACTIVA. For ACTIVA, we used the publicly available pre-trained ACTIVA model to generate synthetic samples. The results show that MAF-FB clearly outperforms ACTIVA across all metrics. MAF-FB achieved better distributional similarity with the real data (WD: 0.43555 vs. 0.79137), more accurate preservation of gene-gene correlations (CD: 0.16031 vs. 0.77847), and better overall alignment between real and synthetic data (MMD: 0.02870 vs. 0.05583). These results highlight the robustness and scalability of the baseline MAF-FB model, which can be effectively used for large-scale single-cell datasets and still outperform state-of-the-art alternatives like ACTIVA.

For the integrated pancreatic dataset, averaged over 5-fold cross-validation, flow-based models again showed strong performance across both distributional similarity metrics and classification outcomes. In terms of WD, MOE-FB achieved the best score among generative models (0.59546), indicating the closest match to the real data distribution. For CD, MAF-FB outperforms others (0.06762), while MOE-FB is still competitive. In the last one, GC has the best MMD (0.002447). Although GC achieved the best MMD score for this dataset, its overall performance across other datasets and metrics was considerably weaker. The classification metrics further highlight the advantage of MOE-FB. It achieved the highest precision (0.9221), recall (0.8935), and F1-score (0.9014) across all models, outperforming both MAF-FB and others.

For HCA-BM10K, averaged over 5-fold cross-validation, FB models lead overall. MOE-FB offers the best downstream performance with the highest recall (0.8802) and F1 (0.8965), while ranking second in precision (0.9308). For the discrepancy metrics CD and MMD, FB models also overtake others: MAF-FB achieves the lowest CD (0.059417) with MOE-FB essentially equal (0.05946), and MOE-FB achieves the lowest MMD (0.005317) with MAF-FB nearly identical (0.005330). CTGAN reaches the lowest WD (0.46898) and is the closest competitor among the SDV library models to the FB models overall.

Original single-cell data contains further biological insights, so it is expected that the synthetic data can indicate biologically similar and meaningful outputs. To assess this, cell-cell interactions were inferred and analyzed for the integrated pancreatic dataset. In Fig 6, heatmaps of cell-cell interactions were generated using ligand-receptor pairs for test and generative model sets. Among the generative models, the number of cell-cell interactions is similar to those of the test set. To validate these outputs, we employed RMSE metric to evaluate the produced error according to test interactions. We compared interaction means, which is the output of CellphoneDB. According to Table 4, MOE-FB has the lowest error rate (0.6522) and followed by MAF-FB with 0.6760. GC has the highest error rate with 0.8740. We also generated dot plots (Fig 7 and S1 Appendix) to visualize the ligand–receptor pairs underlying the examined cell–cell interactions. The identified pairs were consistent between the test data and the synthetic datasets produced by the generative models.

Overall, across integrated pancreatic cohort, PBMC, and HCA-BM10K, flow-based models, especially MOE-FB, consistently lead: MOE-FB offers the best balance of discrepancy metrics and downstream performance, while the baseline MAF-FB is a close second, and FB models still surpass ACTIVA. Among SDV library models, CTGAN is the closest competitor, whereas GC is inconsistent, occasionally strong on MMD but generally weaker on WD, CD, and F1. We examined the integrated pancreatic dataset from a biological perspective by analyzing cell–cell interaction patterns via ligand–receptor dot plots. Among all models, MOE-FB produced similar interactions observed in the test set, yielding the lowest error rate (RMSE:0.6522) from the ground truth. As a result, the evidence indicates that flow-based generators, particularly MOE-FB, offer the most reliable results and performances for single-cell synthesis across datasets and evaluation criteria.

Consequently, we presented a guideline for a generative model-based synthetic data generation and automated cell-type identification, which is our contribution to the knowledge.We developed the FB model with MAF and proposed the MOE-FB model, which combines learnable feature masking and MOE attention with MAF. The potential of FB models is demonstrated by comparing different generative models, especially state-of-the-art ACTIVA. In the future, we also aim to explore pancreatic cancer scaRNA-seq datasets to identify interactions between and potential biomarkers of distinct cell types that make up the tumor microenvironment. Pancreatic cancer calls for a better understanding due to its prevalence, poor prognosis and low survival rates [44,45].

## 4. Materials and methods

### 4.1. Preprocessing datasets

Relatively limited attention is paid to pancreatic tissue in the literature compared to other tissues. To conduct a comprehensive large-scaleanalysis through integrating various datasets, the human pancreas scRNAseq datasets were curated from sources including GEO Repository [46]. Among the datasets frequently used in computational studies, Baron [4], Muraro [5], Segerstolpe [6], and Xin [43] were selected for our large-scaleanalysis. These datasets, which are appropriate for a preliminary work while assessing generative models, were selected as they possess labels/annotations for cells, have a reasonable size, and are widely used. Table 5 displays the technologies and the cell counts.

**Table 5:** Datasets.

Dataset	Technology	Cell
Baron et al year	inDrop	8569
Muraro	CEL-Seq2	2126
Seg	Smart-Seq2	2038
Xin	SMARTer	1600
Total		14333

Pancreatic tissue datasets are accessible through the R packages *scRNAseq* and the *SingleCellExperiment*. SingleCellExperiment [3] has a similar flow to that of *Seurat*. Firstly, the dataset labels were corrected based on the literature [47]. Classes with less than 10 samples/cells were eliminated and “unclear”, “co-expression”, “not applicable”, and “unclassified” cells were removed from the dataset. Cell types that belong to the same cell category but were incorrectly annotated with different labels were merged. Quality control analysis was applied to each dataset individually as follows: First, genes expressed in less than 100 cells and cells expressing less than 100 genes were filtered out. Cells with very high frequencies (doublets, outliers) were eliminated. Then, the mitochondrial frequency of the cells and the external RNA Controls Consortium (ERCC) were examined and cells with high ERCC values were eliminated. However, it is required to examine these values comparatively, cells with low mitochondrial frequency and high ERCC values may be low quality, while cells with high mitochondrial frequency and low ERCC values may be undamaged active cells. After the quality control step, the datasets were individually normalized using sum factors and logarithmic transformation. The common genes of the datasets were kept to enable large-scale analysis for the merged data. Batch effect correction was applied on the integrated data. Then 5 fold CV, 80% training and 20% testing, was carried out for each fold and 3000 HVGs selected using train sets.

We obtained the 68K PBMC dataset from the ACTIVA repository [48]. PBMC68K was originally divided as train and test. We also used PBMC3K, this dataset is publicly accessible from 10x Genomics, and we preprocessed it in the Seurat package tutorials [2]. We divided PBMC3K 80% for training and 20% for testing.

Finally, we employed the HCA human bone morrow (HCA-BM) dataset which is accessible via the HCAData package in Bioconductor [3]. We processed it by using Bioconductor Chapter 42 tutorial. Then, we randomly subsampled 10,000 cells, preserving the original cell type distribution. The numbers per cell type were proportionally determined, and fractional adjustments were distributed across types. Cells were then randomly selected within each type, ensuring proportional representation without oversampling rare populations. We applied 5 fold CV, 80% training and 20% testing for each fold. 3000 HVGs selected using train sets.

### 4.2. Dimensionality reduction and visualization

We used PCA [49], which is one of the most commonly used dimensionality reduction methods. The basic idea underlying the PCA is to find the first principal component with the largest variance in the data and then search for the second component that is uncorrelated with the first component and explain the next largest variance in the same way, and proceed with the identification of the next components similarly. This process is repeated until the new component becomes almost ineffective in terms of contributing to explaining variance in the original data. It consists of the following steps: standardizing the data, creating a covariance matrix, eigenvalue decomposition, and representing the data in the latent space. PCA is commonly used for both dimension reduction and visualization. We used the PCA function from the Python *scikit-learn* library. To maintain comparability with previous studies [11,13,14], we used the 50 dimensional PCA space representation for CD, WD, and MMD metrics evaluation.

Additionally, we used Uniform Manifold Approximation and Projection (UMAP) [50], a non-linear dimensionality reduction technique that constructs a weighted k-nearest neighbor graph in high-dimensional space and optimizes low-dimensional embedding to preserve local and global data structure, for visualization of batch effect on integrated pancreatic dataset.

### 4.3. Synthetic data generation

In scRNAseq data, there is usually an imbalance in sample sizes of distinct cell type categories. There are various approaches to eliminate this problem. Synthetic sample generation is an approach to creating new synthetic samples for classes with fewer samples. The main purpose of generative models is to capture the underlying pattern and structure of the training data and create new synthetic data points that are statistically similar to the original data.

Patki et al. [51] created a synthetic data generation python library called Synthetic Data Vault (SDV) for processing tabular data. It is designed to learn complex dependencies of features in datasets and create synthetic data that preserves these dependencies. Generative models are mostly used for creating images; however, the SDV library was particularly developed to facilitate the use of generative models on tabular data. This library includes CTGAN, TVAE, and GC models and it has not been applied to scRNAseq datasets before. To the best of our knowledge, our study is the first to apply this library to a scRNAseq dataset.

Generative models were employed with the training data, while separate test data was reserved for downstream analyses and cell type classification. This approach ensured that there was no data leakage from the test set during the synthetic data generation process.

In HCA-BM10K and integrated pancreatic datasets, we explored the sample size distribution across classes within the training dataset to determine the optimal number of synthetic samples to generate. The third quartile (Q3) values of the sample size distribution, which equates to 759 for the integrated pancreatic dataset and 670 for the HCA-BM10K dataset, were selected as the target sample size for synthetic data generation. For the classes that contain cells fewer than the corresponding Q3 value, synthetic cells were generated to match the Q3 value. A minimum sample size of Q3 value in each class was ensured, enhancing the robustness of our model against the class imbalance issue. For these two datasets, we executed generative models separately for each cell type by partitioning the data, as the SDV library is limited in its ability to handle high-dimensional samples.

For PBMC3K and PBMC68K, we generated the same number of synthetic samples as in the test set, without considering cell-type labels, in order to benchmark with the ACTIVA model.

#### 4.3.1. Conditional tabular GAN (CTGAN).

ctGAN is a GAN model specifically designed for tabular data. The model learns patterns of the features from the original dataset and generates synthetic data samples that preserve these patterns. GANs [52] consist of two networks that compete with each other: one is a generative network that takes a random noise as input and generates new samples that look like samples from the dataset, and the other one is a discriminator network that tries to distinguish between the real and generated (synthetic) samples. Throughout this comparative training process, the overall model tries to generate more realistic and diverse samples. ctGAN was trained with 100 iterations and other parameters were kept at default values.

#### 4.3.2 Tabular variational autoencoder (TVAE).

TVAE is a VAE model designed for tabular data. VAE models learn to encode input data into a lower-dimensional latent space and then generate new examples by randomly decoding data points from this space. VAE models learn a distribution representing the patterns in the dataset and then generate new samples using these patterns learned. TVAE was run with 100 iterations and other parameters were kept at default values.

#### 4.3.3 Gaussian copula (GC).

GC is a statistical technique used for modeling the dependency structure between variables. Copula models the marginal distributions of individual variables independently and then learns the dependency structure between these independent variables using Gaussian distribution. GC parameters were kept at default values.

#### 4.3.4 Flow-based model (MAF-FB).

FB generative models estimate the exact likelihood of data points, and learn to map data to a latent space and vice versa using invertible transformations. The basic idea of these models [53] is to map a simple distribution, usually a Gaussian distribution, to the target data distribution as close as possible. By applying a series of reversible transformations, these models can capture the gene-gene (i.e., feature) dependencies in the data. *nflows* [54] python library was used and MaskedAffineAutoregressiveTransform (MAF) [55] which is a type of normalizing flow where an autoregressive neural network parameterizes each transformation, was applied. In the nflows library implementation, this network is a Masked Autoencoder for Distribution Estimation (MADE), which enforces the autoregressive structure.

Based on the conceptual description given above, we formulate the flow-based oversampling procedure as follows,

Let $x\;\in \;R^{d}\;$ as data vector. In the flow framework, a sequence of invertible transformations is defined as:


$$h_{\text{0}}=x,\;{\;h}_{l}={\;f}_{l}\left({\;h}_{l-\text{1}}\right)\;(l=\text{1},..\;,L),\;z={\;h}_{L}\;$$ (1)

${\;f}_{\text{0}}(x)$ denotes the identity mapping, representing the input before any transformation.

The latent variable z is required to follow a simple base distribution, typically the standard multivariate Gaussian:


$$z\sim \;N(\text{0},\;I_{d})\;$$ (2)

Masked Affine Autoregressive Transformation:

Each flow layer ${\;f}_{l}$ is implemented as a MAF. In the forward direction (used for likelihood evaluation), the components are transformed sequentially as


$$z_{i}=\frac{x_{i}-\mu_{i}(x_{<i})}{\sigma_{i}(x_{<i})},\;i=\text{1},\;\ldots ,\;d$$ (3)

where $\mu_{i}(.)$ and $\sigma_{i}$(.)>0 are outputs of the masked neural network and depend only on preceding inputs $x_{<i}$

The Jacobian matrix $\frac{\partial_{z}}{\partial_{x}}$ of this transformation is triangular, and its log-determinant is


$$\begin{array}{c} og\left|det\frac{\partial_{z}}{\partial_{x}}\right|=-\sum {\mathrm{\backslash nolimits}}_{i=\text{1}}^{d}log\sigma_{i}(x_{<i}) \end{array}$$ (4)

Log-likelihood:

According to the change-of-variables rule, the data density is


$$logp_{x}(x)=logp_{z}(f_{\text{0}}(x))+log\left|det\frac{{\partial f}_{\text{0}}(x)}{\partial x}\right|$$ (5)

where $f_{\text{0}}$ is the composition of all MAF layers $p_{z}$ is the standard normal density. For ${z=f}_{\text{0}}(x)$, the base log-density is


$$logp_{z}(z)=-\frac{\text{1}}{\text{2}}\left({\left\|z\right\|}_{\text{2}}^{\text{2}}+d\;log(\text{2}\pi )\right)$$ (6)

Training objective (negative log-likelihood minimization), given a dataset ${\left\{x^{\left(n\right)}\right\}}_{n=\text{1}}^{N}$


$$\begin{array}{c} L(\theta )=-\frac{\text{1}}{N}\sum {\mathrm{\backslash nolimits}}_{n=\text{1}}^{N}logp_{x}(x^{\left(n\right)}) \end{array}$$ (7)

We utilized the method presented in [56] for implementation. For hyperparameter optimisation, combinations of the number of layers {1,2,3,4}, the number of hidden features {128, 256, 512, 1024}, and the learning coefficients {1e-2, 1e-4, 1e-6} were tested. The optimal number of layers was identified as 1, the number of hidden features as 1024, the learning coefficient as 1e-6, and the model was executed through 100 iterations. Based on our review of the literature, this is the first study applying a MaskedAffineAutoregressiveTransform-based flow model (MAF-FB) to single-cell RNA-seq data synthesis, offering improved modeling of high-dimensional gene expression patterns.

#### 4.3.5. Mixture of experts flow based model (MOE-FB).

The proposed Mixture-of-Experts Flow-Based (MoE-FB) model is an extension to the flow-based generative modeling with MAF, combining contextual feature masking and mixture-of-experts attention mechanisms. The architecture begins with a Contextual Feature Masking module that adaptively attenuates less informative features using a learnable mask generated from the input.

The Contextual Feature Masking module is implemented as a shallow multilayer perceptron (MLP) that takes the entire feature vector as input and outputs a soft mask indicating the degree of attenuation for each feature. It has two main stages:

(1) Context extraction layer, a fully connected layer that projects the input into a hidden representation, followed by a ReLU activation to capture non-linear interactions.
(2) Mask generation layer – another fully connected layer that maps the hidden representation to the original feature space, followed by a sigmoid activation to produce mask values in the range (0,1).

The mask is learned and applied independently for each input sample; this allows the model to dynamically focus on the most informative features during training.

This masking is followed by an ActNorm layer, which normalizes features and can be selectively enabled or disabled based on a user-defined parameter. ActNorm is applied before both the MoE coupling and MAF block to perform feature-wise normalization.

In each block, the input features are split into two complementary subsets using an alternating binary mask. One feature subset (xa), selected by the binary mask and used to calculate the transformation parameters, is processed by the Mixture-of-Experts (MoE) attention mechanism. This mechanism consists of multiple multi-head attention experts(we selected number of heads as 10 and experts as 4), each capturing different feature dependencies, and a gate network that assigns weights to the experts based on (xa). The aggregated expert output is then used to calculate the scale and shift parameters of an affine coupling transformation applied to the other feature subset (xb). This design preserves invertibility and allows the log-determinant to be calculated exactly from the scale and shift parameters.

After the MOE coupling layer, the two feature subsets are recombined and passed through a Masked Affine Autoregressive Transform. The MAF block enhances the flexibility of the overall transformation and helps capture dependencies that cannot be modeled by the coupling layer alone.

Below, we present the mathematical formulation of the key components in the MoE-FB architecture:

Contextual Feature Masking:

To adaptively mask and smooth sparse features, we generate a mask m ∈ (0, 1)^d derived from the input:


$$c\;=\sigma (W_{m}.\;RELU(W_{c}x\;+b_{c})\;+b_{m}\;$$ (8)

m= 0.5 . c (9)

Where Wc, bc, Wm, bm are learnable parameters (weights and bias) and σ is the sigmoid activation.

The masked input is,


$$\begin{array}{c} x^{\prime }=x\;⊙\;(\text{1}-m)+\;\overset{―}{x}\;*\;m,\;\overset{―}{x}\;=\;\frac{\text{1}}{d}\;\sum {\mathrm{\backslash nolimits}}_{j=\text{1}}^{d}x_{j}\; \end{array}$$ (10)

where ⨀ denotes elementwise multiplication and $\overset{―}{x}\;$ is the mean of a sample’s feature values.

ActNorm:

We apply ActNorm layers and it can be selectively enabled or disabled based on a user-defined parameter,


$$\begin{array}{c} y\;=\;s\;⨀\;x\;+\;b\;,\;log|det\;J|=\sum {\mathrm{\backslash nolimits}}_{j=\text{1}}^{d}log\left|s_{j}\right| \end{array}$$ (11)

Otherwise, the layer is skipped (y=x)

s is learnable scale parameter(feature-wise), b is bias.

MOE Attention Masked Affine Transform:

we split features into two sets using binary mask M ∈ {0, 1}^d^ (coupling partition)


$$x_{a}\;=\;x\;⨀\;M,\;x_{b}=\;x\;⨀\;(\text{1}-M)$$ (12)

Here, $x_{a}$ is the parameter-generating subset and $x_{b}$ is the transformed part. We then process $x_{a}\;$ through a mixture of experts attention module:

For E experts, each attention expert e applies a multi-head attention (MHA) to x_a_


$$h^{\left(e\right)}\;=\;{MHA}^{\left(e\right)}\;(x_{a})$$ (13)

Gating network produces weights


$$g\;=\;softmax(W_{g}.\;RELU(U_{g}x_{a})+b_{g})\;\in \;R^{E}\;$$ (14)

W and b weights and bias, $U_{g}$ projects $x_{a}$ to a hidden representation of size h.

Expert aggregation:


$$\begin{array}{c} h\;=\;\sum {\mathrm{\backslash nolimits}}_{e=\text{1}}^{E}g_{e}*h^{\left(e\right)} \end{array}$$ (15)

Scale and Shift Networks


$$s=tanh(W_{s}h+b_{s})\;(\text{scale})$$ (16)

$$t=W_{t}h+b_{t}\;(\text{shift})$$ (17)

Affine Coupling Transformation:


$$z_{a}=x_{a}$$

$$z_{b}=x_{b}\;⊙\;exp(s)+t$$ (18)

$$z=z_{a}+z_{b}$$

The log-determinant for this transformation is:


$$\begin{array}{c} og\;|det\;J|=\sum {\mathrm{\backslash nolimits}}_{j\in b}s_{j} \end{array}$$ (19)

J is the Jacobian of the transformation.

#### 4.3.6. Automated cell-type-informed introspective variational autoencoder (ACTIVA).

ACTIVA [13] is a deep generative framework specifically designed for single-cell RNA-seq data generation. It is based on a conditional variational autoencoder (cVAE) architecture that integrates a cell-type classifier into the generative process. The framework combines a regularized encoder–decoder structure with an adversarial training strategy, where a discriminator network evaluates synthetic cells to improve generation quality. By leveraging both reconstruction loss and adversarial loss, ACTIVA is able to preserve cell-type–specific gene expression patterns while maintaining diversity in the generated profiles.

### 4.4. Cell type identification

We used labeled/annotated data to overcome the class imbalance issue. Therefore, the RF, which is a frequently preferred and employed supervised learning model [57], was used. A comparative study was conducted to evaluate the classification performance of the experimental setups where synthetic cells generated by generative models.

RF is defined as a forest of decision trees. Decision trees place samples in a hierarchical tree structure from the root node to the leaves. The RandomForestClassifier function was used by using *sklearn* library, and the gain criterion parameter was set to “entropy”. The “n\_estimators” parameter, which specifies the number of trees in the forest, was set to 100. The “max\_depth” parameter specifies the maximum depth of the tree and was set to “none”, which is the default value.

### 4.5. Performance metrics

We evaluated the cell-type classification performance of cells generated by generative models using precision, recall, and F1-score metrics. Additionally, to quantitatively assess the similarity between synthetic and original cells, we employed complementary statistical metrics: Correlation Discrepancy (CD) [13], Wasserstein Distance (WD) [58], Maximum Mean Discrepancy (MMD) [59]. We also used silhouette metric to evaluate batch effect correction and Root Mean Square Error (RMSE) for the comparison of cell-cell interations..

Precision: A metric that measures the ratio of accurately predicted positive samples to all samples predicted as positive. TP: true positives, FP: false positives.


TP/(TP+FP)  (20)

Recall: Measures the ratio of accurately predicted positive samples to all actual positive samples. FN: false negatives


TP/(TP+FN) (21)

F1-measure: A metric that strikes a balance between precision and sensitivity. F1-measure is calculated using the harmonic mean of precision and recall values.


2*Precision*Recall/(Precision+Recall)  (22)

Accuracy: The ratio of correctly predicted samples to the total number of samples. TN: true negatives


(TP+TN)/(TP+TN+FP+FN)  (23)

Correlation Discrepancy (CD): measures pairwise correlation matrices of two sets of data by using Pearson correlation, represented by R. Then, it calculates the differences of two correlation matrices and gets their average value. We used this metric to assess the similarity of synthetic (generated) samples to the original ones.


CD=mean(|R(original)−R(generated)|) (24)

Wasserstein Distance (WD): measures the minimal cost of transforming one probability distribution into another. Lower WD values indicate that the generated samples closely match the real data distribution in terms of overall shape and location. We computed the WD separately for each feature and then obtained the mean value across all features.

Let real data is $X\;\in \;R^{nxd}\;$ and $\tilde{X}\;\in \;R^{mxd}$ is synthetic data. For each feature j, 1D-WD is


$$W_{\text{1}}^{\left(j\right)}=\int_{-\infty }^{\infty }\left|F_{j}(t)-G_{j}(t)\right|dt$$ (25)

Where $F_{j}$ and $G_{j}$ are CDFs(Cumulative Distribution Function) of $X_{j}\;and\;{\tilde{X}}_{j}$

$z_{k}$ are the sorted unique values from both real and synthetic samples of feature j.


$$\begin{array}{c} W_{\text{1}}^{\left(j\right)}=\;\sum {\mathrm{\backslash nolimits}}_{k=\text{1}}^{K-\text{1}}\left|F_{j}(z_{k})-G_{j}(z_{k})\right|\;(z_{k+\text{1}}-z_{k}) \end{array}$$ (26)

Maximum Mean Discrepancy (MMD): measures the difference between two distributions in a reproducing kernel Hilbert space (RKHS). MMD is a kernel-based metric and we use the radial basis function (RBF) kernel. A smaller MMD value indicates that the synthetic and real data distributions are more similar.

Let p is real and q is synthetic data and ϕ represents RBF


$$MMD(p,q;\phi )\;=\;E_{x,x^{\prime }}[\phi (x,x^{\prime })]+E_{y,y^{\prime }}[\phi (y,y^{\prime })-\;\text{2}E_{x,y}[\phi (x,y)]$$ (27)

Three terms are: average similarity within real data, average similarity within synthetic data and minus twice the similarity between real and synthetic data. We obtained average of MMD across several length scales of RBF kernel: 0.005, 0.01, 0.1, 0.5, 1, 2.

Silhouette Score:

We computed silhouette score with respect to batch labels.


$$s(i)=\frac{b(i)\;-\;a(i)}{max\{a(i),b(i)\}}$$ (28)

where a(i) is the mean intra-cluster distance of sample i and b(i) is the mean nearest-cluster distance. The final score is the average over all samples,


$$\begin{array}{c} S\;=\;\frac{\text{1}}{n}\sum {\mathrm{\backslash nolimits}}_{i=\text{1}}^{n}s(i) \end{array}$$ (29)

Batch Silhouette = 1 − |S| (30)

### 4.6. Differentially expressed genes identification

Differential Gene Expression Analysis is performed to detect genes expressed differently across distinct cell types. DEGs demonstrate statistically significant differences in gene expression profiles between cell types. DEGs were detected using Seurat R-package with findAllMarkers function [60]. Statistical test parameter was selected as LR (Logistic Regression). The statistical thresholds are adjusted P-value < 0.05 and average log2 Fold Change(log2FC) > 1. We sorted DEGs according to Log2FC values, and we selected the top 20 differentially expressed genes (DEGs) for each cell type and looked for the mutually exclusive. After selecting the top 20 genes for each cell type, we further refined the lists by removing overlapping genes, ensuring that the final top genes of each cell type were mutually exclusive. Then, we validated the prominent genes against evidence reported in the literature.

### 4.7. Inference of cell-cell interactions and ligand–receptor pairs

Inferring cell-cell communications is essential for understanding several biological processes [61,62]. CellphoneDB is a publicly available tool that identifies ligand-receptor interactions. Cell-cell interactions between cell types are predicted using the Python package CellPhoneDB v5.0.0 [63] with default parameters.

## Supporting information

S1 Table Differantial Expressed Genes for each cell types. (TSV)

S1 Appendix CellphoneDB statistical analysis p-values file (Page 1) Test, (Page 2) MOE-FB, (Page 3) MAF-FB, (Page 4) TVAE, (Page 5) CTGAN, (Page 6) GC. (XLS)

S2 Appendix CellphoneDB statistical analysis means (Page 1) Test, (Page 2) MOE-FB, (Page 3) MAF-FB, (Page 4) TVAE, (Page 5) CTGAN, (Page 6) GC. (XLS)

S3 Appendix Dot plots of CellphoneDB ligand-receptor pairs (A) MOE-FB, (B) MAF-FB, (C) CTGAN, (D) TVAE, (E) GC. (PDF)
