# Supplementary figures and images for: A mixture of attention experts-embedded flow-based generative model to create synthetic cells in single-cell RNA-Seq datasets
Source: PLoS Comput Biol. 2025 Oct 6;21(10):e1013525. doi: 10.1371/journal.pcbi.1013525 (PMC12500167; doi:10.1371/journal.pcbi.1013525)

(A) MOE-FB

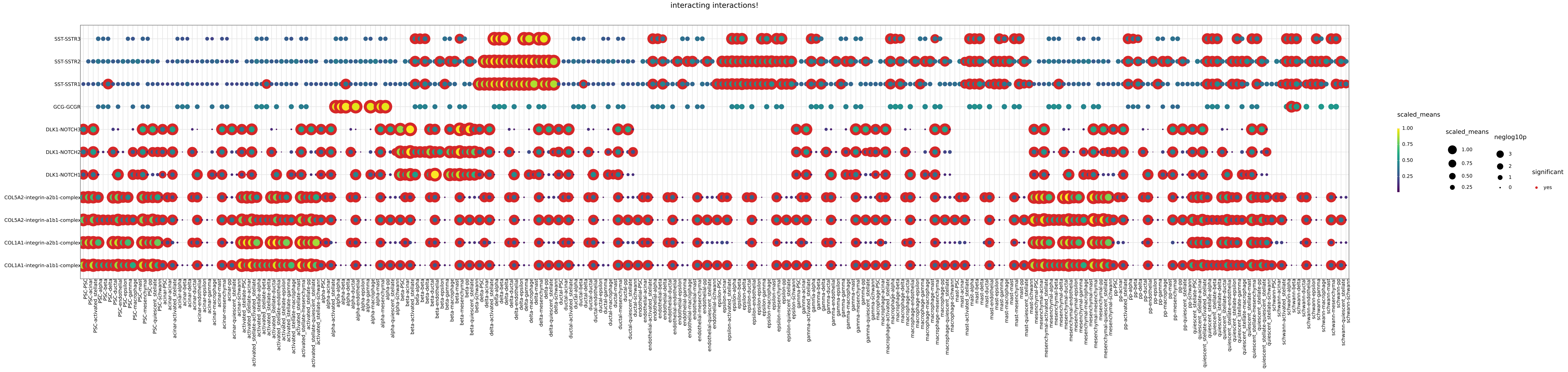

(B) MAF-FB

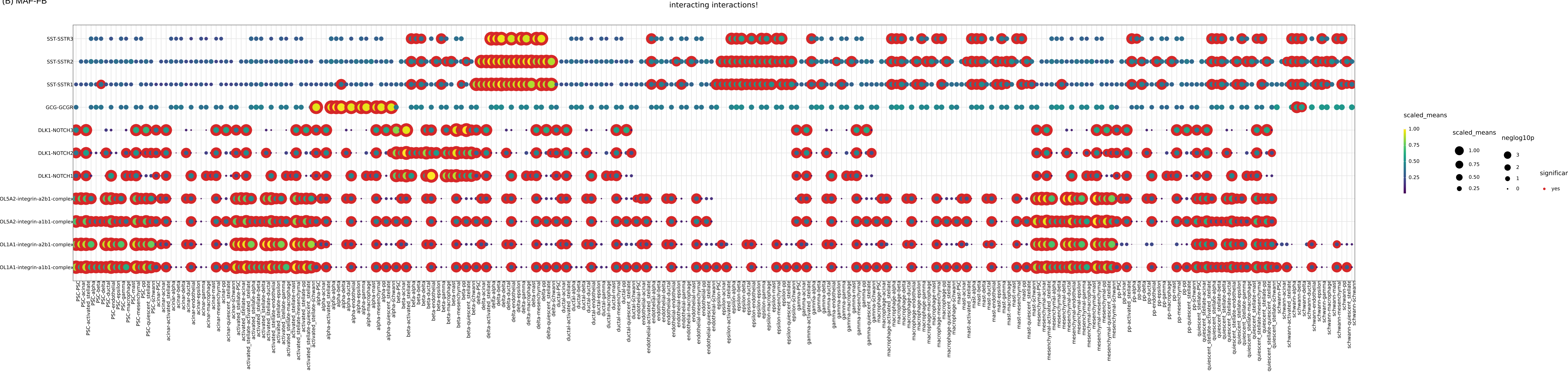

(C) CTGAN

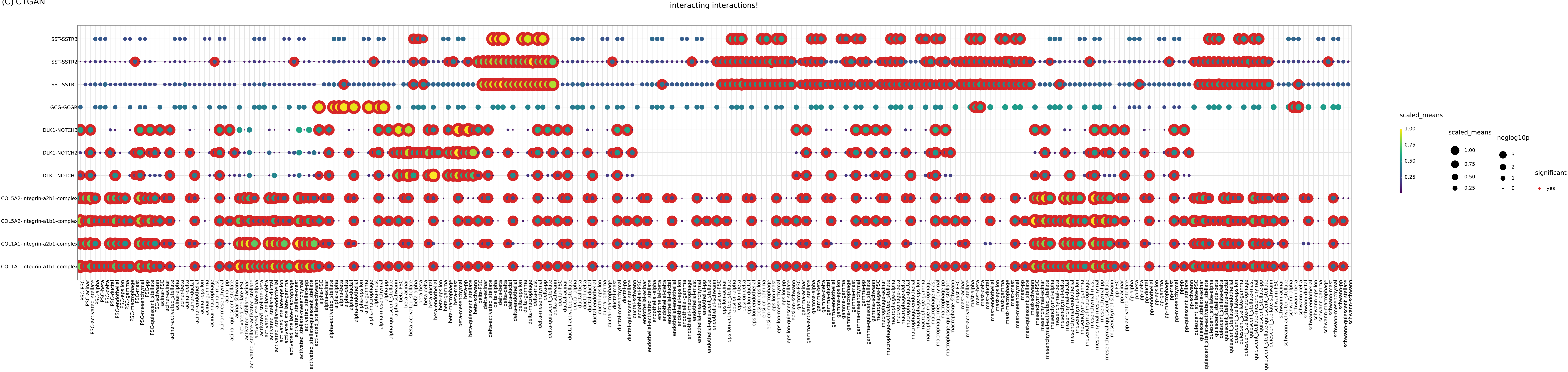

(D) TVAE

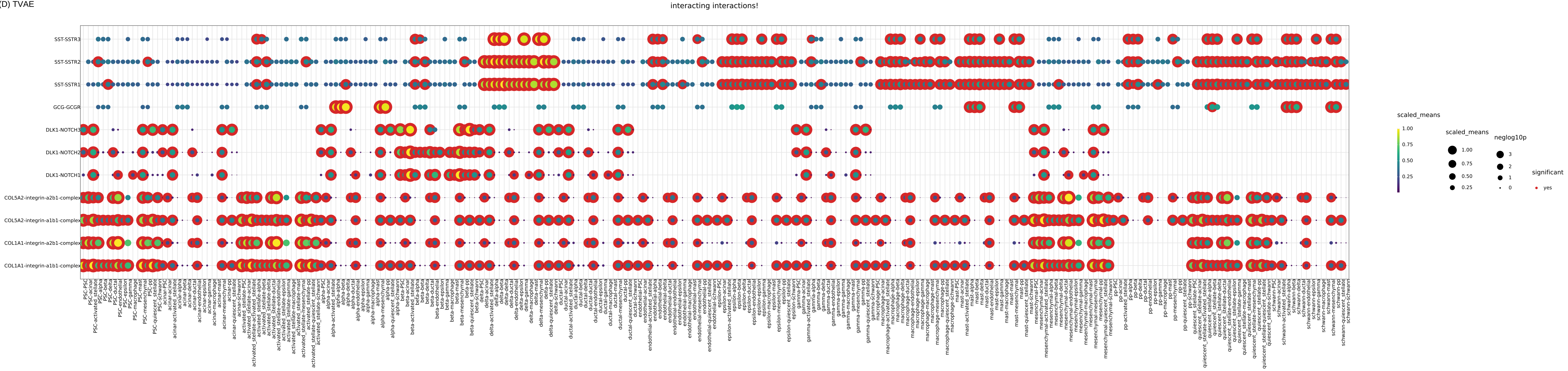

(E) GC

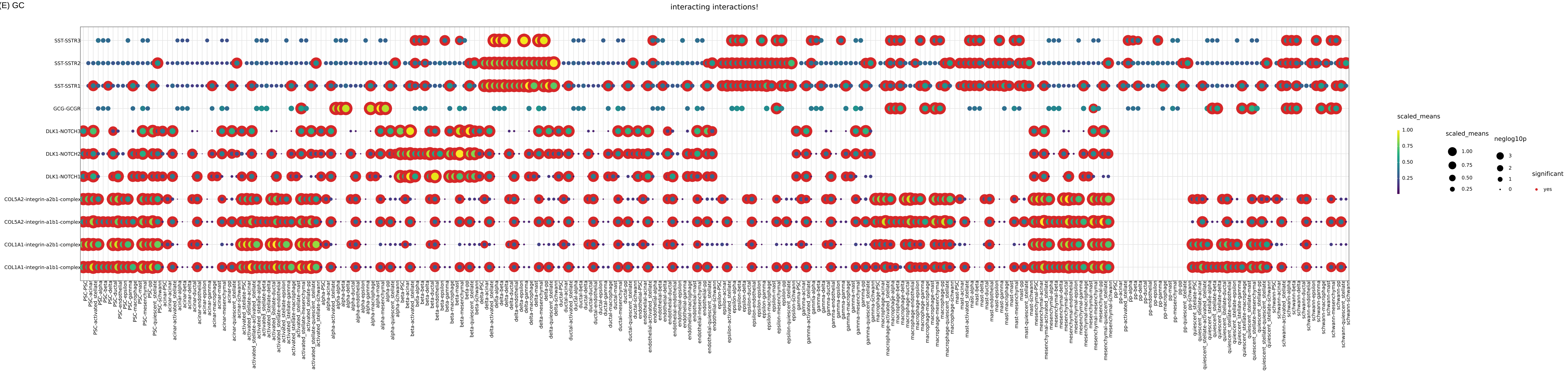

Supplement: S3 Appendix — (PDF) [file pcbi.1013525.s004.pdf]
